# Super-resolution deep learning reconstruction improves brain MRI quality and detection of metastases

**DOI:** 10.1007/s11604-025-01921-3

**Published:** 2025-12-10

**Authors:** Yusuke Asari, Koichiro Yasaka, Jun Kanzawa, Yuki Sonoda, Takahiro Fukushima, Hiroaki Koyama, Saori Koshino, Shigeru Kiryu, Osamu Abe

**Affiliations:** 1 Department of Radiology, Graduate School of Medicine, The University of Tokyo, 7- 3-1 Hongo, Bunkyo-ku, Tokyo, 113-8655 Japan; 2 Department of Radiology, International University of Health and Welfare Narita Hospital, 852 Hatakeda, Narita, Chiba 286 - 0124 Japan

**Keywords:** Magnetic resonance imaging, Deep learning, Image quality, Brain metastasis

## Abstract

**Purpose:**

Accurate identification of brain metastases is critical for determining prognosis and guiding treatment. Deep learning reconstruction (DLR) enhances MRI quality by reducing noise, while super-resolution DLR (SR-DLR) may further improve spatial resolution and lesion detectability. To evaluate SR-DLR versus conventional DLR in detecting and visualizing brain metastases on postcontrast T1-weighted brain MRI.

**Materials and methods:**

This retrospective study included 47 consecutive patients who underwent postcontrast 3D whole-brain T1-weighted MRI between July and December 2024. Images were reconstructed using both SR-DLR and DLR. Three independent readers evaluated metastatic lesion detection and rated image quality. Subjective assessments included lesion visibility, visibility of normal structures, sharpness, noise, and overall image quality. Objective metrics—full width at half maximum (FWHM), edge rise distance (ERD), edge rise slope (ERS), signal-to-noise ratio (SNR), and contrast-to-noise ratio (CNR)—were also measured. Statistical tests included jackknife alternative free-response receiver operating characteristic (JAFROC) analysis, Wilcoxon signed-rank test, McNemar’s test, and paired *t*-tests, with significance threshold of *p* < 0.050.

**Results:**

A total of 117 brain metastases were detected in 47 patients (mean age, 59 years ± 18; 27 men). SR-DLR demonstrated significantly better lesion detection performance than DLR (mean figure of merit = 0.842 vs. 0.797; *p* = 0.042). Subjective image quality ratings favored SR-DLR for lesion and structure visibility, sharpness, noise, and overall quality in most cases. Objectively, SR-DLR yielded lower FWHM (1.2 mm vs. 1.9 mm; *p* < 0.001), higher ERS (791.3 mm^− 1^ vs. 645.3 mm^− 1^; *p* = 0.013) indicating enhanced sharpness as well as improved CNR (27.5 vs. 24.9; *p* < 0.001) compared to DLR.

**Conclusion:**

Compared to DLR, SR-DLR significantly enhances brain MRI quality and improves detection of metastatic lesions.

## Introduction

Brain metastases are a common manifestation of malignant tumor spread and are frequently seen in patients with primary lung and breast cancers [1, 2]. Their presence or absence has a significant impact on prognosis, making imaging-based detection critically important [3]. Precise lesion quantification is also essential in evaluating eligibility for stereotactic radiotherapy [4–6]. Contrast-enhanced magnetic resonance imaging (MRI) is regarded as a highly effective modality for detecting brain metastases [7, 8]. However, small metastatic lesions can sometimes be missed, underscoring the need for improved detection performance [9, 10].

Recent advances in deep learning have shown considerable promise across multiple applications, including radiological imaging [11]. In diagnostic imaging, deep learning reconstruction (DLR) has been developed to enhance image quality by reducing noise through deep learning algorithms [12–15]. A more recent development, super-resolution DLR (SR-DLR), offers additional improvements in spatial resolution alongside noise reduction [16–18]. SR-DLR has shown effectiveness in visualizing small anatomical structures such as cranial nerves and cerebral microbleeds in brain MRI [19, 20]. Since brain metastases often appear as small lesions, SR-DLR may provide advantages in their detection. However, to date, no prior study has utilized SR-DLR for the detection and depiction of brain metastatic lesions.

This study aims to compare the performance of SR-DLR and conventional DLR in detecting and visualizing brain metastases on MRI.

## Materials and methods

### Data collection

**Fig. 1:**
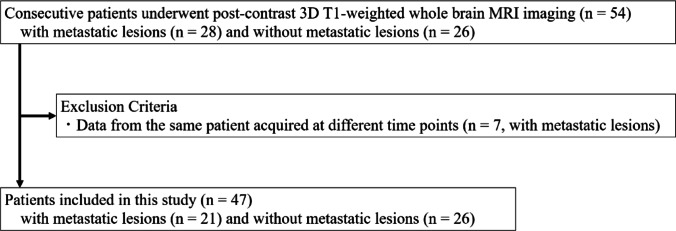
Patient inclusion

This retrospective study was approved by the institutional review board, which waived the requirement for informed consent. Data were obtained from patients who underwent postcontrast 3D T1-weighted whole-brain MRI using a 3T scanner between July and December 2024 (Fig. 1). To avoid data redundancy, only the first examination from the same patient performed at different times was included; subsequent examinations were excluded.

### MRI protocol

MRI was conducted using a 3T scanner (Vantage Centurian; Canon Medical Systems, Otawara, Japan). A gadolinium-based contrast media (Gadovist, Bayer, Leverkusen, Germany) was administered intravenously at a dose of 0.1 ml/kg. Axial 3D postcontrast T1-weighted MP-RAGE images were obtained using the following imaging parameters: repetition time, 7.3 ms; echo time, 2.0 ms; flip angle, 10°; echo train length, 192; acquisition matrix, 256 × 256; and pixel bandwidth, 391 Hz. The parallel acquisition technique (SPEEDER, Canon Medical Systems, Otawara, Japan) was applied in-plane with a parallel reduction factor of 2.49. Compressed sensing was not utilized. The acquisition time for each case was 152 s. Image reconstruction was performed using two methods: SR-DLR (Precise IQ Engine; Canon Medical Systems) and conventional DLR (Advanced Intelligent Clear-IQ Engine; Canon Medical Systems). The reconstructed image parameters for SR-DLR/DLR were as follows: slice thickness, 1.0 mm for both; pixel spacing, 0.3333 mm for SR-DLR and 0.5000 mm for DLR; and matrix size, 768 × 768 for SR-DLR and 512 × 512 for DLR. For the up-sampling stage of SR-DLR, zero-filling is applied to the k-space with a three-fold interpolation factor to improve spatial resolution. No post processing was applied in viewers.

### Lesion detection

Dataset randomization was performed by a radiologist with 16 years of experience. The process of creating the reference standard for identifying brain metastases involved two independent reviewers: the radiologist who randomized the dataset above and a radiology resident with 4 years of experience. They initially analyzed the images and identified potential lesions. During this phase, they had access to additional resources such as past and future images, as well as images of different sequences from the same day (e.g., diffusion-weighted images). Afterward, each reviewer presented their draft reference standard, and any discrepancies between the drafts were addressed through a thorough review of the images, radiology reports, and electronic medical records. The final reference standard was then determined through this collaborative process, ensuring it was comprehensive and validated by multiple data sources. Three readers—two board-certified radiologists with 9 and 7 years of experience and a radiology resident with 4 years of experience—independently evaluated the images. This radiology resident reader, with 4 years of experience, is different from the one who established the reference standard. For each suspected brain metastasis, they recorded the location and assigned a confidence level using a 4-point scale: 4, definitely present; 3, probably present; 2, possibly present; and 1, not present. Image viewing and interpretation were performed using ImageJ software (https://imagej.net/ij/). The evaluations were conducted in two separate sessions, with randomization ensuring that SR-DLR and DLR images from the same patient were not presented in the same session. The randomization process occurred in two stages. First, patients whose SR-DLR images were included in Session 1 were randomly selected, and the DLR images of the remaining patients were assigned to Session (1) Other images were included in Session (2) Next, randomization was performed both within Session 1 and within Session 2, which included SR-DLR and DLR images. To minimize bias, readers were blinded to both the type of reconstruction (SR-DLR or DLR) and any clinical information about the patients. A minimum 1-week washout period was maintained between sessions to reduce recall bias, ensuring proper separation of the evaluations.

### Subjective image quality assessment

The same three readers involved in lesion detection independently assessed the image quality of both SR-DLR and DLR images in a second randomized session, held at least 1 week after the lesion detection session. For cases with brain metastases, lesion visibility was rated on a 4-point scale: 4, clearly visible; 3, mildly blurred; 2, considerably blurred; and 1, not visible. In patients with multiple lesions, all lesion sizes were measured, and the lesion with the median size was selected for assessment. If there was an even number of lesions, the smaller of the two median-sized lesions was chosen. Additionally, all cases were evaluated for visualization of normal anatomical structures (cerebral superficial veins and falx cerebri), image sharpness, image noise, and overall image quality, using the following scales:

● Anatomical structure visualization: 4, clearly visualized; 3, mildly blurred; 2, considerably blurred; and 1, blurred to a degree that impacts diagnostic interpretation.

● Sharpness: 4, very sharp; 3, moderately sharp; 2, slightly sharp; and 1, not sharp enough for diagnostic purposes.

● Noise: 4, minimal to no noise; 3, mild noise; 2, considerable noise; and 1, noise level hampers diagnostic accuracy.

● Overall image quality: 5, excellent; 4, better than standard; 3, standard; 2, below standard; and 1, poor.

### Objective image quality assessment

**Fig. 2:**
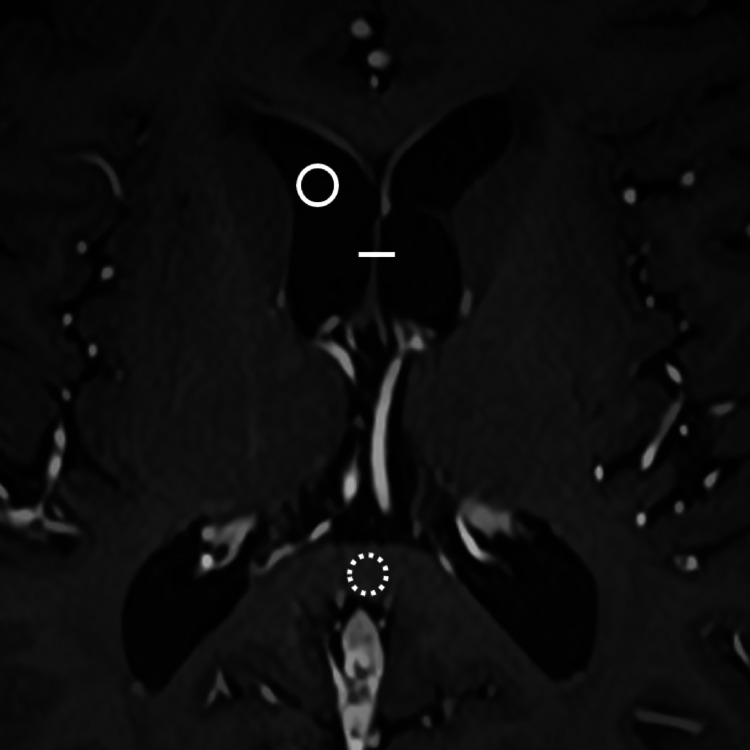
A linear ROI was placed along the septum pellucidum (line). Circular ROIs were positioned on the right anterior horn of the lateral ventricle (circle) and the splenium of the corpus callosum (dotted circle). ROI, region of interest

Objective image assessment was conducted by a radiology resident with 4 years of experience using ImageJ software. Identical positions and sizes of linear and circular regions of interest (ROIs) were applied to both SR-DLR and DLR images (Fig. 2). The images of both SR-DLR and DLR were displayed on screen simultaneously and “Sync Windows” plugin of ImageJ was used to co-register ROIs between reconstruction. For the linear ROIs, a 3–5 mm line was placed across the septum pellucidum. From the resulting intensity profiles, the full width at half maximum (FWHM, mm), edge rise distance (ERD, mm), and edge rise slope (ERS, mm⁻¹) were computed. ERD and ERS values were averaged from both edges. Lower FWHM and ERD values correspond to greater sharpness, while higher ERS values improved image sharpness.

For the circular ROIs, 3–5 mm diameter circles were positioned in the right frontal horn of the lateral ventricle and the midline splenium of the corpus callosum, ensuring that no visible lesions were included. The mean signal intensity and standard deviation within each ROI were used to derive the following metrics:

● SNR_BRAIN = SI_BRAIN/SD_BRAIN

● SNR_CSF = SI_CSF/SD_CSF

● CNR = (SI_CSF − SI_BRAIN)/√((SD_BRAIN² + SD_CSF²)/2)

Here, SNR_BRAIN and SNR_CSF represent the signal-to-noise ratios for brain tissue and cerebrospinal fluid, respectively. CNR denotes the contrast-to-noise ratio. SI and SD refer to the mean signal intensity and standard deviation within the ROI. The rationale for choosing the septum pellucidum and splenium of the corpus callosum for SNR and CNR measurement lies in their signal homogeneity and minimal contamination from surrounding structures, which provide reliable assessment of imaging quality. For cases with metastatic lesions, quantitative analyses for the evaluation of brain metastasis visualization were also performed. Circular ROIs (3–5 mm in diameter) were placed in the representative lesion used for the Subjective Image Quality Assessment and in normal brain parenchyma. For small lesions, care was taken to ensure that the ROI did not exceed the lesion boundary. The contrast ratio between the lesion and the brain, as well as the CNR, were calculated according to the equation described above.

### Statistical analysis

All statistical analyses were conducted using R (version 4.3.1). Comparisons between patients with and without brain metastases were performed using the *t*-test and Fisher’s exact test. Detection performance and confidence levels were evaluated using jackknife alternative free-response receiver operating characteristic (JAFROC) analysis (https://cran.r-project.org/web/packages/RJafroc/index.html), implemented with the R package “RJafroc.” The figure of merit (FOM) in RRRC (random reader random case) setting, which is comparable to the area under the curve in conventional ROC analysis, served as the performance metric. For sensitivity analysis per reader, lesions with a confidence score of 2 or higher were included, and comparisons were made using McNemar’s test. Size-stratified sensitivities were also calculated for three groups: lesions measuring < 5 mm in diameter, between 5 mm and < 10 mm, and 10 mm or larger. The Wilcoxon signed-rank test was used to compare subjective image quality scores, while paired *t*-tests were applied to assess objective image quality parameters and false positives per case. No multiple comparison correction was applied. A *p*-value < 0.050 was considered statistically significant.

## Results

### Patient characteristics

Of the 54 MRI datasets initially collected, 7 were excluded due to duplication, resulting in 47 datasets included in the final analysis. Among these, 21 patients were found to have at least 1 brain metastasis, while 26 patients showed no evidence of brain metastases. Patients with metastatic lesions were older (67.8 ± 11.5 years) compared to those without lesions (52.5 ± 19.1 years, *p* = 0.002). There was no significant difference in sex distribution between the lesion and nonlesion groups (Table 1). The primary tumor sites in the patients were as follows: lung (*n* = 13), esophagus (*n* = 4), and others (*n* = 4). A total of 117 brain metastases were identified: 68 lesions measured < 5 mm in diameter, 35 measured between 5 mm and < 10 mm, and 14 were 10 mm or larger. The lesion counts per case were distributed as follows: 7 cases had 1 lesion, 2 cases had 2 lesions, 3 cases had 3 lesions, 1 case had 4 lesions, 2 cases had 6 lesions, 1 case had 7 lesions, 1 case had 8 lesions, 1 case had 12 lesions, 2 cases had 15 lesions, and 1 case had 24 lesions.

**Table 1:** Patient demographic characteristics in the metastatic lesion and nonlesion groups

	Metastatic lesion group (*n* = 21)	Nonlesion group (*n* = 26)	*p*-value
Age (years, mean ± standard deviation)	67.8 ± 11.5	52.5 ± 19.1	0.002*
Sex (male, female)	13, 8	14, 12	0.767

Age, *t*-test. Sex, Fisher’s exact test.

*Statistically significant difference

### Lesion detection

The FOM values for SR-DLR were 0.872 for Reader 1, 0.868 for Reader 2, and 0.785 for Reader 3, all of which were significantly higher than those for DLR (Reader 1, 0.833; Reader 2, 0.831; Reader 3, 0.727; *p* = 0.042, Table 2). Sensitivities with SR-DLR were 0.786 for Reader 1, 0.915 for Reader 2, and 0.692 for Reader 3. For DLR, the sensitivities were 0.812, 0.872, and 0.650, respectively. The differences in sensitivity per reader between SR-DLR and DLR were not statistically significant in either the total lesion analyses or the size-stratified analyses (Table 3). Sensitivity per case was same between DLR and SR-DLR (1.00 for Readers 1 and 2, and 0.952 for Reader 3). Specificity per case was 0.778, 0.704, and 0.778 for Readers 1–3 with DLR, and 0.815, 0.815, and 0.852 with SR-DLR. The number of false positives per reader was lower with SR-DLR (Reader 1, 72; Reader 2, 133; Reader 3, 39) compared to DLR (Reader 1, 95; Reader 2, 166; Reader 3, 71). The number of false positives per case was lower with SR-DLR (Reader 1, 0.532; Reader 2, 0.894; Reader 3, 0.234) compared to DLR (Reader 1, 0.660; Reader 2, 1.043; Reader 3, 0.468), with statistical significance observed for Reader 3 (*p* = 0.020).

**Table 2:** Lesion detection outcomes

	SR-DLR	DLR	*p*-value	95% CI of mean difference
Reader1	0.872	0.833		
Reader2	0.868	0.831		
Reader3	0.785	0.727		
Mean	0.842	0.797	0.042*	( 0.002, 0.088 )

Figures of merit derived from the reader’s diagnostic confidence scores are presented using jackknife alternative free-response operating characteristic analysis. CI, confidence interval; DLR, deep learning reconstruction; SR-DLR, super-resolution deep learning reconstruction

*Statistically significant difference

**Table 3:** Sensitivity score of lesion detection

	Reader	SR-DLR	DLR	*p*-value
Overall	1	0.786 (92/117)	0.812 (95/117)	0.677
	2	0.915 (107/117)	0.872 (102/117)	0.267
	3	0.692 (81/117)	0.650 (76/117)	0.499
Size				
< 5 mm	1	0.750 (51/68)	0.750 (51/68)	1.000
	2	0.912 (62/68)	0.853 (58/68)	0.289
	3	0.603 (41/68)	0.529 (36/68)	0.424
5 mm≤, and < 10 mm	1	0.857 (30/35)	0.886 (31/35)	1.000
	2	0.914 (32/35)	0.886 (31/35)	1.000
	3	0.800 (28/35)	0.800 (28/35)	1.000
10 mm≤	1	0.786 (11/14)	0.929 (13/14)	0.48
	2	0.923 (13/14)	0.923 (13/14)	1.000
	3	0.857 (12/14)	0.857 (12/14)	N/A

DLR, deep learning reconstruction; SR-DLR, super-resolution deep learning reconstruction

### Subjective image quality assessment

**Fig. 3:**
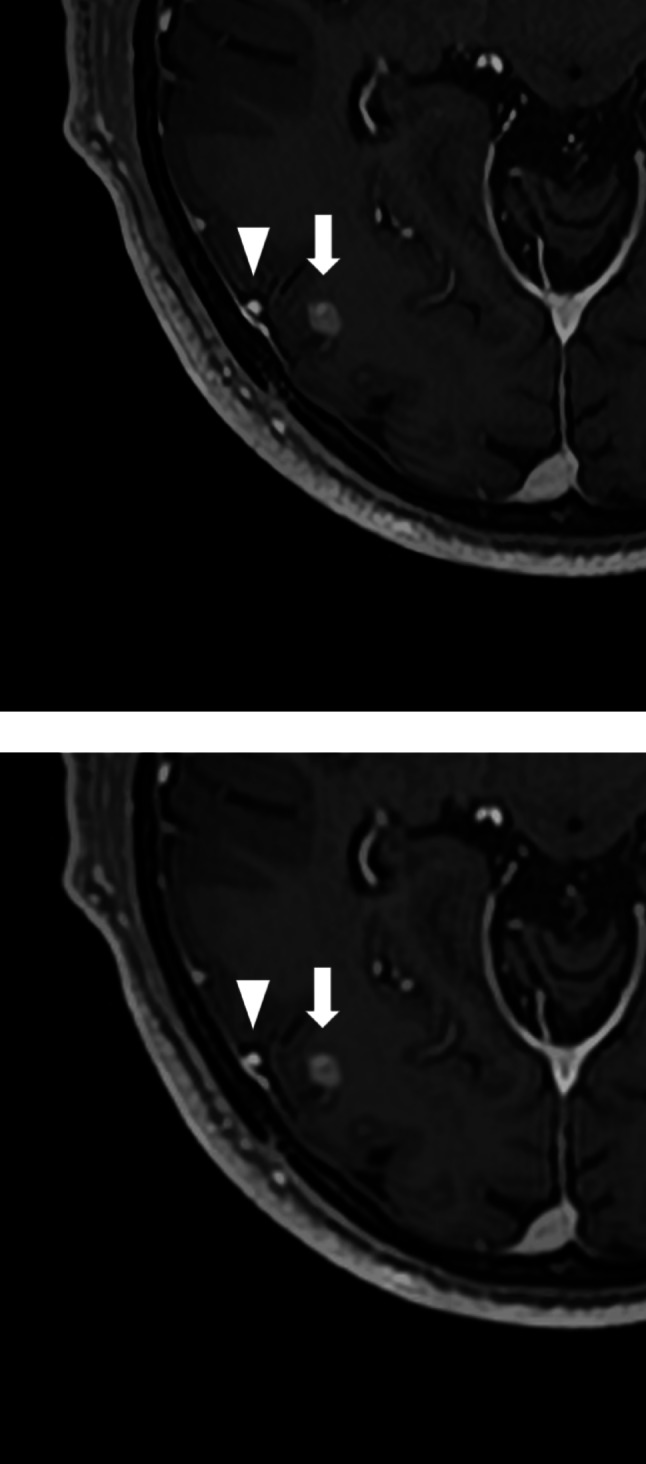
3D postcontrast T1-weighted MRI acquired using super-resolution deep learning reconstruction **a** and deep learning reconstruction **b**, showing a metastatic lesion (arrow) and a cerebral vein (arrowheads) in an 84-year-old male patient

**Fig. 4:**
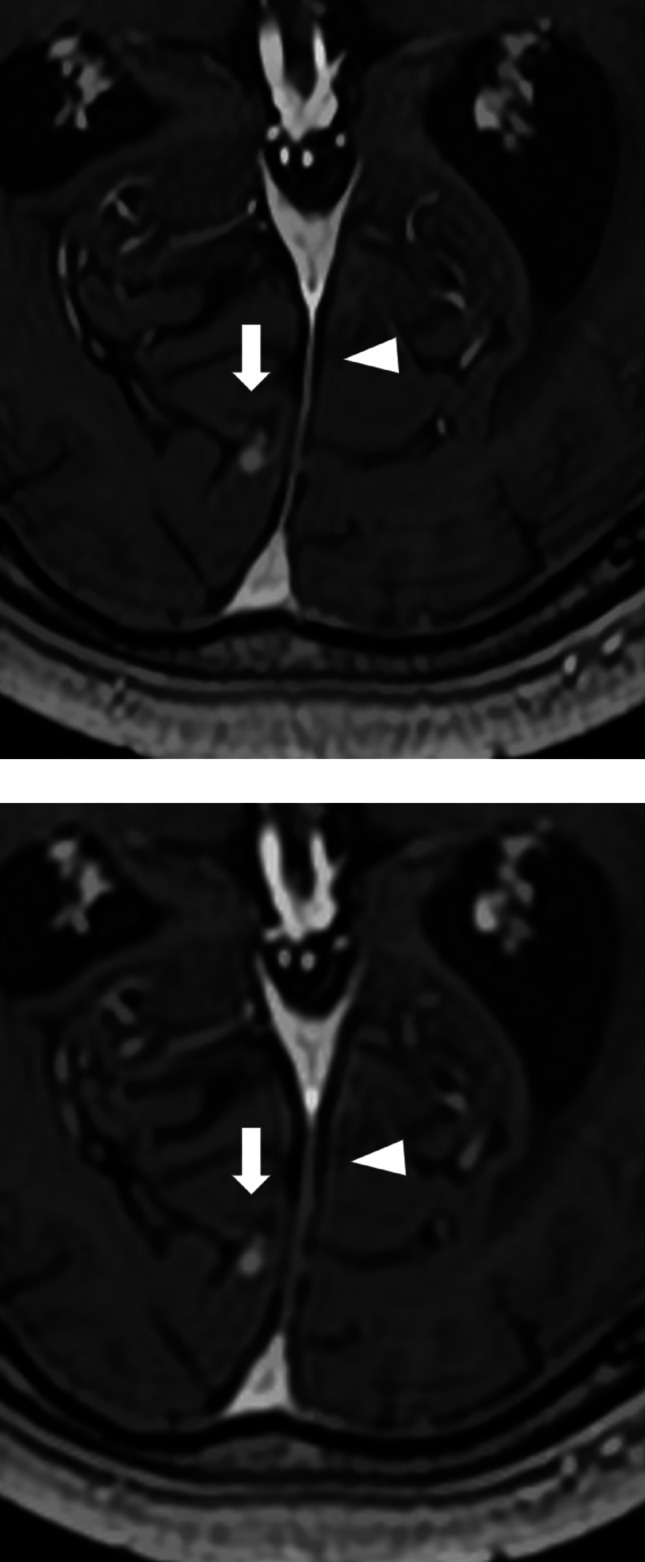
3D postcontrast T1-weighted MRI acquired using super-resolution deep learning reconstruction **a** and deep learning reconstruction **b**, depicting a metastatic lesion (arrow) and the falx cerebri (arrowheads) in a 75-year-old male patient. In the lesion detection analysis, Reader 3 failed to detect this lesion with DLR but successfully detected it with SR-DLR

**Fig. 5:**
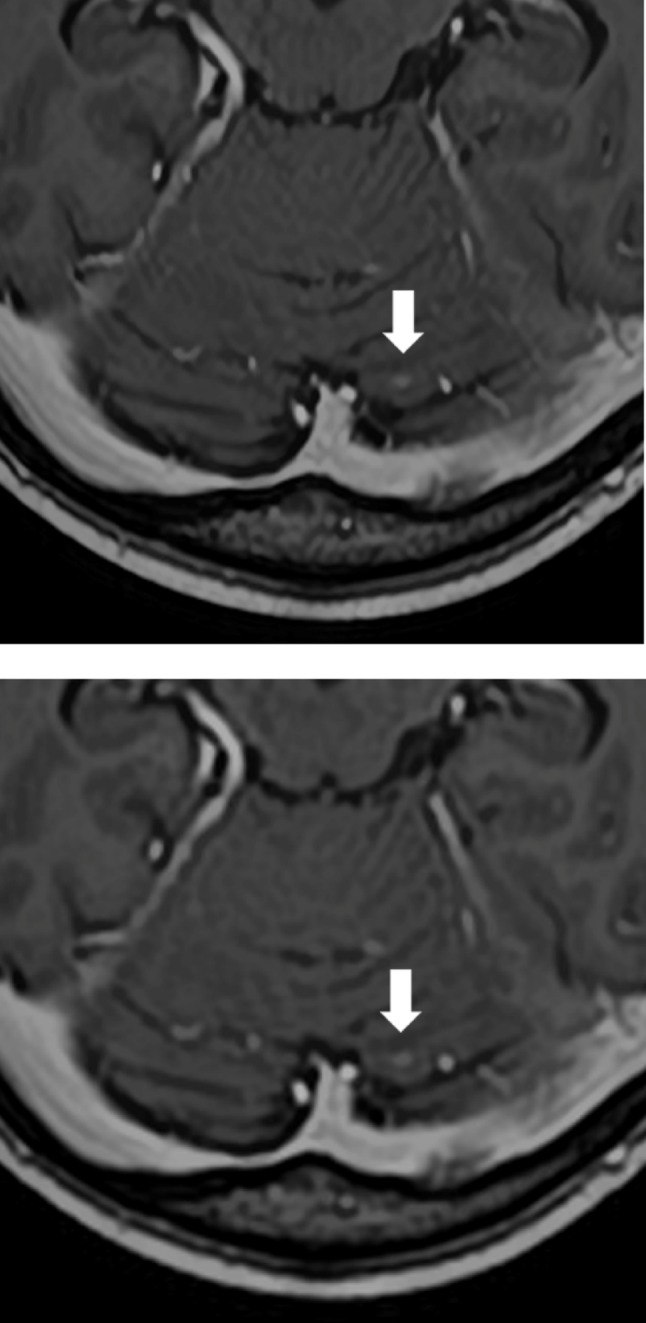
3D postcontrast T1-weighted MRI acquired using super-resolution deep learning reconstruction **a** and deep learning reconstruction **b**, depicting a metastatic lesion (arrowhead) in a 70-year-old female patient. In the lesion detection analysis, Reader 2 failed to detect this lesion with DLR but successfully detected it with SR-DLR

All three readers gave higher ratings to SR-DLR for the visualization of metastatic lesions, with Reader 2 showing a statistically significant preference. For the depiction of normal structures and image sharpness, Readers 2 and 3 favored SR-DLR, while Reader 1 found no significant difference between the two methods. In terms of image noise, Readers 1 and 3 rated SR-DLR as having less noise. Overall image quality was rated highly by all readers for SR-DLR (Figs. 3, 4 and 5; Table 4).

**Table 4:** Subjective image quality assessment results

	Reader	SR-DLR	DLR	*p*-value
Metastasis	1	6/11/4/0	6/11/3/1	1
	2	14/5/2/0	5/10/6/0	0.003*
	3	3/12/5/1	3/11/6/1	0.821
Superficial vein	1	44/3/0/0	47/0/0/0	0.149
	2	44/3/0/0	35/12/0/0	0.003*
	3	32/13/2/0	16/18/13/0	< 0.001*
Falx	1	47/0/0/0	46/1/0/0	1
	2	45/2/0/0	27/20/0/0	< 0.001*
	3	35/10/2/0	15/23/8/1	< 0.001*
Sharpness	1	44/3/0/0	44/3/0/0	1
	2	44/3/0/0	26/21/0/0	< 0.001*
	3	38/8/1/0	24/18/5/0	0.002*
Image noise	1	43/4/0/0	34/13/0/0	0.008*
	2	42/5/0/0	38/9/0/0	0.182
	3	30/16/1/0	17/26/4/0	0.003*
Overall image quality	1	33/5/6/1/2	25/8/11/1/2	0.028*
	2	40/5/1/1/0	28/17/2/0/0	0.008*
	3	13/23/6/2/3	5/18/17/6/1	0.004*

The number of patients assigned to each rating score (5/4/3/2/1 or 4/3/2/1) is reported. DLR, deep learning reconstruction; SR-DLR, super-resolution deep learning reconstruction

*Statistically significant difference

### Objective image quality assessment

In the linear ROI analysis, SR-DLR demonstrated significantly shorter FWHM and higher ERS compared to DLR. In the round ROI analysis, SR-DLR showed significantly higher SNR_CSF and CNR than DLR. No significant differences were found for ERD and SNR_BRAIN (Table 5). For cases with metastatic lesions, SR-DLR demonstrated better CNR between the lesion and the brain compared to DLR with statistical significance (Table 6).

**Table 5:** Objective image quality assessment results of normal brain

	SR-DLR	DLR	*p*-value	95% CI of mean difference
FWHM (mm)	1.2 ± 0.4	1.9 ± 0.8	< 0.001*	( −1.0, −0.5 )
ERD (mm)	1.7 ± 0.5	1.7 ± 0.4	0.578	( −0.3, 0.1 )
ERS (mm^− 1^)	791.3 ± 406.8	645.3 ± 287.6	0.013*	( 32.4, 259.5 )
SNR_BRAIN	33.9 ± 11.0	33.0 ± 13.3	0.327	( −1.0, 2.9 )
SNR_CSF	13.1 ± 5.1	11.0 ± 4.1	< 0.001*	( 1.0, 3.1 )
CNR	27.5 ± 7.0	24.9 ± 6.7	< 0.001*	( 1.4, 3.8 )

CI, confidence interval; CNR, contrast-to-noise ratio; DLR, deep learning reconstruction; ERD, edge rise distance; ERS, edge rise slope; FWHM, full width at half maximum; SNR_CSF, signal-to-noise ratio for CSF; SNR_BRAIN, signal-to-noise ratio for the brain; SR-DLR, super-resolution deep learning reconstruction

*Statistically significant difference

**Table 6:** Objective image quality assessment results with metastatic lesion

	SR-DLR	DLR	*p*-value	95% CI of mean difference
CNR	8.2 ± 3.7	6.1 ± 3.1	< 0.001*	( 1.1, 3.1 )
Contrast ratio	1.6 ± 0.3	1.5 ± 0.3	0.191	( 0, 0.1 )

CI, confidence interval; CNR, contrast-to-noise ratio; DLR, deep learning reconstruction; SR-DLR, super-resolution deep learning reconstruction

*Statistically significant difference

## Discussion

This study compared SR-DLR and DLR in detecting and visualizing brain metastases on postcontrast T1-weighted brain MRI. The findings indicate that SR-DLR provides superior performance in lesion detection, image quality, and overall diagnostic evaluation compared to DLR. Specifically, SR-DLR yielded higher FOM values across all readers, highlighting its advantage in lesion detection. This is clinically meaningful, as accurate identification of brain metastases is essential for determining prognosis and guiding treatment decisions [21]. The differences in sensitivity between SR-DLR and DLR were not significant for any reader. Sensitivities with SR-DLR were higher for Readers 2 and 3, while sensitivity with DLR was higher for Reader 1. The disparity might be attributed to individual visual biases and preferences, or to familiarity with the neuroimaging subspecialty, as Reader 1 was the most experienced in terms of years. This variability in reader performance suggests that the generalizability of these findings may be limited.

In terms of subjective image quality, SR-DLR also outperformed DLR. All three readers assigned higher scores to SR-DLR for metastatic lesion visualization, with Reader 2’s preference reaching statistical significance. Two readers also favored SR-DLR for normal structures visualization and sharpness, whereas one reader noted no significant difference, possibly reflecting variability in reader experience. Objective image quality analysis further supported SR-DLR’s superiority, showing significantly better sharpness, as reflected by FWHM and ERS values. The enhanced visualization of brain metastases and small anatomical features, such as the cerebral superficial veins and falx cerebri, likely contributed to SR-DLR’s improved performance in lesion detection.

In terms of image noise, all readers subjectively rated SR-DLR as having lower noise levels, with two evaluations reaching statistical significance. SR-DLR also showed significantly better objective results for both SNR_CSF and CNR. Prior studies evaluating SR-DLR in brain MRI for detecting microbleeds did not report reduced noise levels compared to DLR [20]. This discrepancy may be due to differences in imaging protocols, as earlier studies used 2D MRI, while the present study utilized 3D MRI. In 3D imaging, thinner slices and smaller voxel volumes generally result in lower SNR, potentially enhancing the observable benefits of SR-DLR.

The integration of SR-DLR into real-world clinical practice could significantly enhance the management of brain metastases, particularly in the context of follow-up imaging and treatment response assessment. With its superior performance in lesion detection and image quality, SR-DLR could improve clinicians’ ability to monitor small metastatic lesions. This would be particularly valuable for detecting early progression or recurrence of metastases, enabling timely interventions. Moreover, SR-DLR’s ability to provide clearer visualization of normal brain structures and reduce image noise could aid in more accurate treatment planning, such as radiation therapy, where precision is crucial.

Although SR-DLR demonstrated clear advantages over DLR in lesion detection and image quality, several study limitations must be considered. The relatively small sample size, single-vendor and single-institution setting, and retrospective research design may limit the generalizability of the findings. Additionally, factors such as the magnetic field strength, coil configuration, and sequence family that could influence the results were not tested. Recent brain metastasis literature highlights sequence-level differences in imaging performance, such as the use of motion-sensitized driven-equilibrium-enhanced 3D T1 turbo spin-echo sequences, which have been shown to improve lesion contrast and detection sensitivity compared to traditional sequences [22–25]. Therefore, the choice of imaging protocol, including these sequence variations, could affect the applicability of our findings to other clinical settings. Furthermore, the influence of SR-DLR on clinical outcomes, such as treatment planning and patient survival, remains to be determined. Further research involving larger, multi-center, longitudinal studies, incorporating varying imaging protocols and field strengths, is needed to assess the long-term impact of SR-DLR in managing brain metastases. In addition, since neither surgery nor autopsy were commonly performed, it was difficult to assess whether all the suspected lesions were truly brain metastases. Therefore, it cannot be ruled out that bias may have been introduced during the preparation of the reference standard.

In conclusion, this study demonstrates the superior performance of SR-DLR compared to DLR in the detection and visualization of brain metastases on postcontrast T1-weighted brain MRI. SR-DLR provided significant improvements in lesion detection and image quality, supporting its potential role in enhancing clinical decision-making.

## Data Availability

The data that support the findings of this study are available on request from the corresponding author.
